# Health-Care Costs, Glycemic Control and Nutritional Status in Malnourished Older Diabetics Treated with a Hypercaloric Diabetes-Specific Enteral Nutritional Formula

**DOI:** 10.3390/nu8030153

**Published:** 2016-03-09

**Authors:** Alejandro Sanz-Paris, Diana Boj-Carceller, Beatriz Lardies-Sanchez, Leticia Perez-Fernandez, Alfonso J. Cruz-Jentoft

**Affiliations:** 1 Nutrition Unit, Universitary Hospital Miguel Servet, Isabel the Catholic 1-3, Zaragoza 50009, Spain; dianna_bc@hotmail.com (D.B.-C.); bealardies@gmail.com (B.L.-S.); leticca@hotmail.com (L.P.-F.); 2 Geriatric Department, Universitary Hospital Ramón y Cajal, Ramón y Cajal de Sanitary Investigation Institution (IRYCIS), Madrid 28034, Spain; ajcruzjentoft@telefonica.net

**Keywords:** diabetes enteral formula, diabetes mellitus, malnutrition, heath care cost

## Abstract

Diabetes-specific formulas are an effective alternative for providing nutrients and maintaining glycemic control. This study assesses the effect of treatment with an oral enteral nutrition with a hypercaloric diabetes-specific formula (HDSF) for one year, on health-care resources use, health-care costs, glucose control and nutritional status, in 93 type-2 diabetes mellitus (T2DM) malnourished patients. Changes in health-care resources use and health-care costs were collected the year before and during the year of intervention. Glucose status and nutritional laboratory parameters were analyzed at baseline and one-year after the administration of HDSF. The administration of HDSF was significantly associated with a reduced use of health-care resources, fewer hospital admissions (54.7%; *p* < 0.001), days spent at hospital (64.1%; *p* < 0.001) and emergency visits (57.7%; *p* < 0.001). Health-care costs were reduced by 65.6% (*p* < 0.001) during the intervention. Glycemic control (short- and long-term) and the need of pharmacological treatment did not change, while some nutritional parameters were improved at one year (albumin: +10.6%, *p* < 0.001; hemoglobin: +6.4%, *p* = 0.026). In conclusion, using HDSF in malnourished older type-2 diabetic patients may allow increasing energy intake while maintaining glucose control and improving nutritional parameters. The use of health-care resources and costs were significantly reduced during the nutritional intervention.

## 1. Introduction

Diabetes mellitus (DM) is a major health problem due to its prevalence, mortality and cost. The International Diabetes Federation estimated in 2014 its global prevalence at 8.3% [1]. Moreover, it imposes a large and growing economic burden on the health-care system and society [2,3,4] due to the increase in the number of people with diagnosed diabetes, the increased frequency of chronic complications and the wider application of new and expensive technologies and treatments [5].

Disease-related malnutrition is common in diabetic patients [6]. It is present in 21.2% of hospitalized older diabetics [7]. Hospital related malnutrition is associated with treatment intolerance, poor prognosis, increased hospital-acquired infections, poor wound healing and longer hospitalizations [8].

Diabetes-specific enteral nutritional formulas are postulated as effective alternatives for nutritional treatment in diabetic subjects, being associated with maintenance of glycemic control [9,10,11], due to their content of slowly digested and absorbed carbohydrates and monounsaturated fats [9]. Nevertheless, the long-term benefits on glycemic control or the economic impact of such formulas are unclear [6,12]. For a product intervention to represent a good value, it should not only be efficacious but also be worth the scarce resources that were given up to purchase it [13].

The objective of this study is to assess the effect of enteral supplementation with the hypercaloric diabetes-specific formula (HDSF) Glucerna^®^ 1.5 Cal, from Abbott Nutrition, on the use of health-care resources, health-care costs, glucose control (short- and long-term) and nutritional status in type-2 diabetes mellitus (T2DM) older malnourished patients in a real life setting.

## 2. Materials and Methods

### 2.1. Design

An observational, retrospective study of computerized databases of outpatient medical records from the Nutrition outpatient clinic of the “Universitary Hospital Miguel Servet” of Zaragoza, Spain, was performed. All patients diagnosed with T2DM plus protein-caloric malnutrition living in the community who were started on an oral nutritional supplement between 2011 and 2013 in our outpatient clinics and had at least one follow up visit after completion of treatment were included. Those who were unable to move to the clinic, as well as patients with cancer, renal or liver insufficiency or degenerative diseases, were not included in the study. Furthermore, those with recent acute illness, recent surgery or recent hospitalization were excluded, as such situations have a confounding effect on plasma albumin levels. Malnutrition was defined as weight loss > 10% and baseline albumin < 3.5 g/dL. Diabetes was defined according to usual diagnostic criteria. Subjects were prescribed two daily servings of 220 mL for a year of the HDSF Glucerna^®^ 1.5 Cal, from Abbott Nutrition, composed of 20% protein, 35% carbohydrates and 45% fat (Supplemental Table S1).

### 2.2. Recorded Variables

For each patient, sociodemographic and clinical variables, glucose status, nutritional laboratory parameters, health-care resources use associated with the treatment of diabetes and health-care costs were collected during the year before and after the prescription of the HDSF. Health-care costs included hospitalization costs associated with the treatment of diabetes and the HDSF treatment overrun cost. Hospitalization costs (Spanish € 2014) were calculated by the “Universitary Hospital Miguel Servet” billing department for each of the patients included in the study. All prices are expressed both in euros and converted to US $ 2015 [14]. The HDSF treatment overrun cost was calculated by subtracting the HDSF (€0.0204/kcal) ($0.0279) from the standard formula cost (€0.0128/kcal) ($0.01786), based on the Spanish National Health System current maximum financed prices [15]. Considering that both formulas consist of 1.5 kcal/mL and that each patient received 440 mL daily, the HDSF administered cost was €4,914.36 ($6,858.40) per year while the same amount of a standard enteral formula would have cost €3,083.52 ($4,303.31) per year.

### 2.3. Cost Per Controlled Patient

In the consensus statement published by the American Diabetes Association, and the European Diabetes Association in 2012, the recommended glycemic goal for patients with low life expectancy or multiple comorbidities is an HbA1c between 7.5% and 8.0% [16]. In addition, an albumin level < 3.5 g/dL has been shown to be a good predictor of malnutrition in older individuals [17]. Based on these, two endpoints were defined for the present analysis to represent effective control of glycaemia (HbA1c ≤ 7.5% or 8.0%) and nutrition (Albumin ≥ 3.6 g/dL). The cost per controlled patient was defined as the cost (mean hospitalization cost after the instauration of the nutritional treatment plus the cost of the HDSF) of a patient achieving each of the endpoints over one year [18].

### 2.4. Correlation and Regression Analysis

To relate the use of resources with the HDSF administration, a correlation analysis was performed to measure the degree of association between pairs of variables, according to Pearson’s (parametric variables) or Spearman’s (non-parametric variables) tests. The change in use of health-care resources (hospitalizations and emergency department visits) and health-care costs (hospitalization costs and hospitalization cost plus HDSF overrun cost) were correlated with the change in glycemic control variables (glucose and HbA1c) and the change in laboratory parameters (albumin, creatinine, total high density lipoprotein (HDL) and low density lipoprotein (LDL) cholesterol, triglycerides, iron, ferritin, hemoglobin, lymphocytes, vitamin B_9_ and vitamin B_12_).

Multivariate regression analysis was used to identify if changes in the main glycemic (glucose or HbA1c) and nutritional (albumin or hemoglobin) parameters could predict changes in the health-care costs due to the establishment of the enteral nutrition.

### 2.5. Statistical Analysis

SPSS v.20 was used for data analysis. The statistical level considered significant was *p* ≤ 0.05. Absolute and relative frequencies for categorical variables and measures of central tendency and dispersion (mean, standard deviation-SD) for quantitative variables were calculated. Normality of quantitative variables was assessed using the Kolmogorov-Smirnov test. Variation of variables before and after the prescription of the HDSF was calculated with Student’s *t* and Wilcoxon’s tests for parametric and non-parametric variables, respectively.

### 2.6. Ethical Statement

All data recorded in this study were managed following the confidentiality and no traceability conditions were established in the Spanish Organic Law on Personal Data Protection. The protocol of the study was accepted by the Spanish Medicines and Sanitary Products Agency and approved by the “Universitary Hospital Miguel Servet” Clinical Research Ethics Committee.

## 3. Results

Data from 93 patients were included in the study. Mean (SD) age was 84.9 (10.8) years, 48.4% were males. Most of the subjects (75.3%) were living at home, and 22.6% lived in nursing homes. Mean (SD) BMI was 23.55 (3.16) kg/cm^2^ and mean (SD) time since the diagnosis of T2DM was 7.6 (4.8) years. Before starting oral supplementation, mean (SD) HbA1c was 6.62% (1.44%) and subjects were treated with metformin (26%), insulin glargine (19%), sulfonylureas (17%) or other oral drugs. One out of four (23%) received no drug treatment. Most subjects (91%) presented at least one comorbidity, hypertension (80%), dyslipidemia (63%) and heart diseases (51%) being the most common. Complications of diabetes were present in roughly one of five (neuropathy 22%, nephropathy 18%, and retinopathy 18%). Mean Charlson index was 4.8 (1.6), with 44% of the sample having values between four and five, defining high load of comorbidity (Table 1).

### 3.1. Use of Health-Care Resources and Health-Care Costs

**Figure 1:**
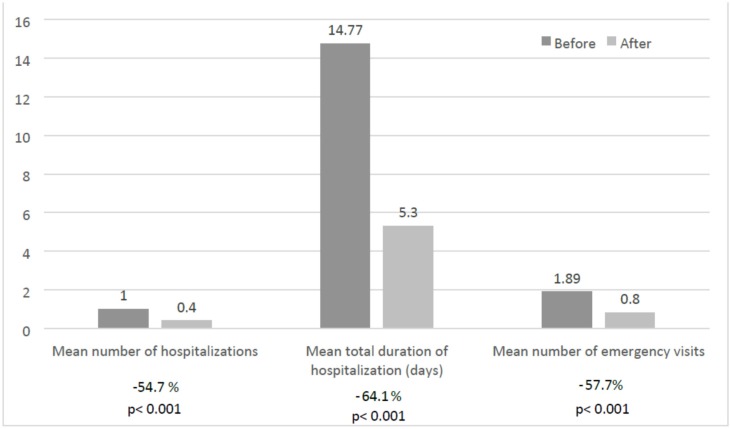
Health-care resources consumption before and after treatment with the HDSF. The reduction in health-care resources consumption after HDSF is expressed as percentage and included under the bars.

The sample health-care resources consumption was significantly reduced after the instauration of the HDSF. The mean (SD) number of hospital admissions decreased significantly from 1.0 (1.2) in the year before intervention to 0.4 (0.8) in the year of nutrition intervention (54.7% decrease, *p* < 0.001). The same happened with the number of days in hospital, falling from 14.77 (22.73) to 5.30 (12.94) days (64.1% drop, *p* < 0.001). The number of visits to the emergency department also decreased from 1.89 (1.68) to 0.80 (0.95) (57.7%, *p* < 0.001) (Figure 1).

**Figure 2:**
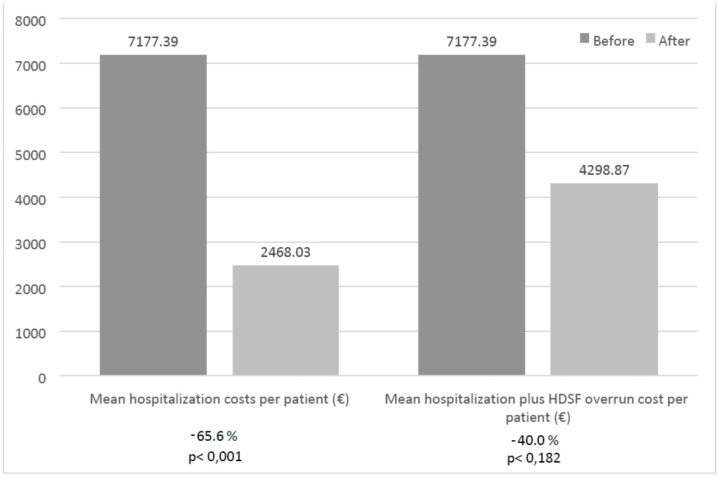
Hospitalization cost before and after treatment with HDSF. Mean hospitalization costs per patient are expressed as €. The percentage of cost reduction after HDSF is expressed as percentage and included under the bars.

In addition, gross hospitalization costs were also significantly reduced from a mean ± (SD) of €7,177 ± €11,700.57 ($10,016.10 ± $16,329.12) to €2,468 ± €6,270.84 ($3,444.30 ± $8,751.48) per patient (65.6% per patient, *p* < 0.001). If the overrun of using an HDSF instead of a standard formula (€1,830.84) ($2,555.09) is added to the hospitalization cost after the enteral nutrition, this cost would still be reduced by 40%, from a mean (SD) of €7,177 ± €11,700.57 ($10,016.10 ± $16,329.12) to €4,298.87 ± €6,270.84 ($5,999.43 ± $8,751.48) (*p* < 0.182) (Figure 2).

### 3.2. Glucose Control Status

The supplementation with HDSF maintained glycemic control. Glucose decreased non-significantly from a mean of 129 mg/dL to 123 mg/dL, while HbA1c was 6.62% at baseline and 6.45% (*p* = 0.143) after nutrition intervention (Table 2). Moreover, the percentage of patients with HbA1c levels ≤ 7.5% increased after the nutritional treatment from 69.9% to 78.5%.

### 3.3. Nutritional Laboratory Parameters

After the supplementation with HDSF, there was an improvement in most of the sample nutritional parameters. Albumin and hemoglobin significantly increased by 10.6%, from 3.12 to 3.45 mg/dL and by 6.4%, from 11.35 g/dL to 12.08 g/dL, respectively. The lipid profile also improved, being that the HDL increased 2.4%, while the LDL and triglycerides decreased 2.4% and 8.9%, respectively. Finally, ferritin values diminished 35.7%, while vitamins B_9_ and B_12_ increased 12.9% and 27.1%, respectively (Table 2).

### 3.4. Cost Per Controlled Patient

Of the 93 patients included in the study, 22 (23.7%) achieved the first composite endpoint (HbA1c < 8.0% and albumin > 3.6 g/dL) and 20 (21.5%) reached the second composite endpoint (HbA1c ≤ 7.5% and albumin ≥ 3.6 g/dL). The cost per controlled patient reaching the first and second composite endpoints was €31,207.39 ($43,552.50) and €34,328.13 ($47,907.76), respectively.

### 3.5. Correlation and Regression Analysis

To assess the relation between the use of resources and the HDSF administration, changes in use of health-care resources and health-care costs were correlated with changes in glycemic control variables and with changes in laboratory parameters.

A statistically significant (*p* < 0.05) direct correlation was found between the variation in HbA1c and the change in hospitalization length, hospitalization costs and hospitalization plus HDSF overrun costs and between variations in glucose and the change in the number of emergency visits. Increments in HbA1c were associated with longer hospitalizations and increased costs and greater glucose values were associated with higher number of emergency visits.

Variations on the hemoglobin and lymphocyte count were indirectly correlated with the variation in hospitalization length and cost, being that an increment in the hemoglobin value or in the lymphocyte count was associated with lesser hospitalizations length and cost. Statistically significant correlations (*p* < 0.05) are presented in Table 3.

A multivariate regression analysis to assess whether changes in nutritional and glycemic clinical parameters could explain changes in hospitalization costs associated with the treatment of diabetic malnourished patients was performed, considering the latter as the dependent variable. Results showed that an appropriate improvement in hemoglobin levels may predict a reduction in hospitalization expenses (regression coefficient −3167.2; *p* = 0.010).

## 4. Discussion

Diabetes mellitus places a considerable burden on patients in terms of morbidity and mortality and on the society in terms of costs [19]. Diabetic patients are at risk of both malnutrition [11] and hospital readmissions [20], often requiring nutritional support [10]. Enteral HDSF have been specially developed for the treatment of malnourished diabetic patients, as well as for maintaining glycemic control [10,11,21,22] which, if not managed, can have profound consequences on clinical outcomes [23].

This study explored the effect of an HDSF on the use of health-care resources, health-care costs, glycemic control and nutritional status when prescribed to treat malnutrition in older diabetic patients. Results show that the administration of the HDSF was associated with less use of health care resources. There was a health-care costs reduction, even if the excess cost of the formula (compared with formulas used to treat malnourished non-diabetic patients) is taken into account. Excess hospitalization and longer hospital stays can be seen as quality indicators of diabetes care [24]. Ordoñez *et al*. [25] reported that nutritional status influenced the length of stay and clinical outcomes in hospitalized patients. Hamdy *et al*. [6] analyzed clinical variables and real cost data from 125,000 hospital inpatient episodes over a 10-year period, further confirming that the use of “glycemia-targeted specialized nutrition” on malnourished diabetics significantly improved health-care efficiency reducing the length of hospitalizations and episodes costs compared to standard formulas, where the cost of the billable glycemia-targeted or standard product was included.

In line with these findings and despite HDSFs being more expensive than standard formulas, a net global saving in hospitalization costs was found in the present analysis. These savings in hospitalization costs can be considered paramount given the exponential increment on health-care costs observed after the age of 50 years [26] and to the extra costs associated with malnutrition (up to €5,829 ($8,134.85) per patient) [27]. Moreover, the indirect cost savings associated with the decrease in hospitalizations, the economic benefits related to avoiding insulinizations or the possible improvement in patients’ and carers’ Health-Related Quality of Life (HRQoL) have not been considered, which could translate into additional benefits.

Our results showed that patients’ glucose and HbA1c were unchanged with nutritional intervention, even with a large increase in caloric intake (up to 660 kcal/day with optimal compliance) needed in a malnutrition setting. In addition, the percentage of patients with HbA1c ≤ 7.5%, an indicator of good glycemic control in older patients [16], increased with the nutritional support, avoiding new patient insulinization or a change in the glucose-lowering drug treatment. Other publications have shown similar findings. The systematic review of Elia *et al.* [11] showed that DSFs improved glycemic control, compared to standard formulas. Lansink *et al.* [21] published a study with T2DM patients receiving either specific or standard formula, finding that the HDSF did not affect fasting glucose levels and contributed to improving glycemic control. Finally, the systematic review published by Ojo *et al.* [10] concluded that HDSF was more effective controlling glucose profiles, including postprandial glucose, HbA1c and insulinemic response. However, this is the first study that shows the effect of HDSF on patients’ glycemic control during one-year follow up.

Some laboratory parameters related to malnutrition were improved, with significant increases in albumin and hemoglobin after intervention. Although some have cautioned against using albumin as a measure of nutritional status due to its association with other clinical phenomena such as inflammation [28]. In situations of clinical stability in older people living in the community, albumin could be a good marker of nutritional status [17]. Moreover, anemia has been found to be associated with hypoalbuminemia and malnutrition in geriatric populations [29].

Other nutritional variables such as creatinine, HDL, lymphocytes and vitamins B_9_ and B_12_ were also enhanced, although the differences were not significant. Nonetheless, the lack of statistical significance does not necessarily mean absence of clinical relevance [30], indicating that the general improvement in nutritional status in the context of malnutrition should be taken under consideration.

Despite its higher acquisition charge, the costs per controlled patient (with two different definitions, HbA1c < 8.0%/albumin > 3.6 g/dL and HbA1c < 7.5%/albumin > 3.6 g/dL) with HDSF were €31,207.39 ($43,552.50) and €34,328.13 ($47,907.76), respectively. Although the values are not comparable, a reasonable threshold for the willingness to pay for each quality adjusted life years according to National Institute for Health and Care Excellence (NICE) guidelines would be within a range of £20,000 ($29,412) to £30,000 ($44,118) [31], giving an idea of the value for money obtained from implementing the treatment with HDSF. This cost estimation offers the advantage of trading off the cost and effect of the HDSF and is suitable for a short time horizon such as the one used in this analysis, where standardized benefit outcomes as quality-adjusted life years are less appropriate. However, this approach does not provide any willingness to pay contexts and constitutes an outcome measure specific for malnourished diabetic patients [18].

Significant relationships were found between the improvement in HbA1c, glucose parameters and hemoglobin, and the decrease in health-care resources use and health-care costs, indicating that the control of glycemic parameters and the improvement in general nutritional status is related to hospitalization cost and health-care resources spent reduction. Moreover, a multivariate regression demonstrated that hemoglobin improvement can predict lower health-care costs. Hemoglobin has been proposed as a valuable marker of nutritional status [32]. Serum levels of hemoglobin are a biochemical indicator routinely used by clinicians to monitor nutritional status in chronically critically ill patients [33]. In this sense, a negative and significant but weak correlation between hemoglobin (as a nutritional indicator) and the duration of patient hospital stays has been previously demonstrated [34] in hospitalized adult and elderly patients.

This study has limitations common in observational retrospective database studies, such as the inability to control for data not captured. The use of each subject as its own control may allow for other not controlled confounding factors to partially explain the results, as changes in health condition and in utilization of health care resources after initation of HDSF may be related to the supplement or to other concomitant factors. To reduce this potential risk, acute diseases or surgical procedures before HDSF prescription were ruled out because of their potential confounding effect on malnutrition diagnostic criteria. Furthermore, no evident change in approach to care or in providers after initiation of HDSF happened in any subject that was performed in a single center and by a single team, the only nutrition team in the area.

Compliance with the intervention could not be properly assessed. However, the main nutritional parameters were available for the majority of patients (albumin accessible for 100% and HbA1c for 90.3% of the patients). More studies including safety variables related to glycemic control, such as hypoglycemias, should be performed to explore other outcomes associated to HDSF, although the fact that changes in the pharmacologic treatment were not necessary might suggest that hypoglycemias were not frequent in this population.

Finally, the presented information should be analyzed in its context since the data included represent a specific population; however, we feel that this is an interesting contribution to the literature on the health economic benefit of diabetes specific formulations for malnourished patients with T2DM.

## 5. Conclusions

In conclusion, the present study provides a picture of the clinical and economic benefits associated with the use of HDSF for the management of T2DM malnutrition. HDSFs are capable of maintaining long-term glucose control, reducing the negative effects of prolonged glycemic fluctuations.

## Figures and Tables

**Table 1:** Patients’ characteristics.

**(A) Sociodemographic and Clinical Variables**	**Patients**
Mean age (SD)	84.9 (10.8)
Mean BMI (SD)	23.55 (3.16)
Mean time since the diagnosis of T2DM (SD)	7.6 (4.8)
Mean Charlson index (SD)	4.8 (1.6)
Males	48.4%
Living at home	75.3%
Living at nursing homes	22.6%
**(B) Pharmacological Treatment**	
Metformin	26%
Dietetic treatment	23%
Insulin glargine	19%
Sulfonylureas	17%
Mixed insulin	8%
DPP4 inhibitors	7%
**(C) Comorbidities**	
Hypertension	80%
Dyslipidemia	63%
Heart Disease	51%
Brain disease	40%
Neuropathy	22%
Nephropathy	18%
Retinopathy	18%

**Table 2:** Sample clinical variables mean values before and after the prescription of the enteral nutrition.

Variable	Before			After			Dif. (%)	*p*-Value
	*N*	Mean	SD	*N*	Mean	SD		
Glucose (g/dL)	93	128.98	60.87	93	122.78	56.65	−4.8	0.428
HbA1c (%)	84	6.62	1.44	84	6.45	0.99	−0.17	0.143
Albumin (g/dL)	93	3.12	0.34	93	3.45	0.46	10.6	**0.000**
Creatinine (mg/dL)	92	1.05	0.69	78	1.21	1.18	15.2	0.307
Cholesterol (mg/dL)	89	168.43	62.80	76	172.30	72.95	2.3	0.462
LDL (mg/dL)	62	101.08	30.07	53	98.70	33.43	−2.4	0.492
HDL (mg/dL)	67	42.82	16.59	54	43.85	15.39	2.4	0.451
Triglycerides (mg/dL)	69	144.19	93.75	54	131.33	55.45	−8.9	0.163
Iron (μg/dL)	60	52.70	35.63	57	47.60	24.51	−9.7	0.503
Ferritin (ng/dL)	59	267.94	356.97	55	172.24	185.75	−35.7	0.322
Hemoglobin (g/dL)	64	11.35	2.17	59	12.08	1.80	6.4	**0.026**
Lymphocytes (%)	64	21.85	10.89	57	23.54	9.48	9.5	0.326
Vitamin B_9_ (pg/mL)	48	9.74	5.87	40	11.00	7.13	12.9	0.073
Vitamin B_12_ (pg/mL)	47	347.26	230.51	40	441.28	399.28	27.1	0.412

**Table 3:** Statistically significant correlations between quantitative variables.

Before and After Variation	Hospitalization Length	Emergency Visits	Hospitalization Costs	Hospitalization + HDSF Overrun Costs
HbA1c	0.273		0.271	0.268
Glucose	-	0.233	-	-
Hemoglobin	−0.369	-	−0.364	−0.364
Lymphocytes	−0.350	-	−0.349	−0.349

## Supplementary Materials

The following are available online at http://www.mdpi.com/2072-6643/8/3/153/s1, Table S1: HDSF composition.

## Abbreviations

The following abbreviations are used in this manuscript:

HDSF	hypercaloric diabetes-specific formula
DSF	diabetes-specific formula
T2DM	type-2 diabetes mellitus
DM	Diabetes mellitus
SD	standard deviation
BMI	body mass index
DPP4	Dipeptidyl Peptidase IV
HRQoL	Health Related Quality of Life
HbA1c	glycosylated hemoglobin
