# Supplementary material for: Health-Care Costs, Glycemic Control and Nutritional Status in Malnourished Older Diabetics Treated with a Hypercaloric Diabetes-Specific Enteral Nutritional Formula
Source: Nutrients. 2016 Mar 9;8(3):153. doi: 10.3390/nu8030153 (PMC4808881; doi:10.3390/nu8030153)
Supplement: Supplementary file 1 [file nutrients-08-00153-s001.docx]

Supplementary Materials: Health-Care Costs, Glycemic Control and Nutritional Status in Malnourished Older Diabetics Treated with a Hypercaloric Diabetes-Specific Enteral
Nutritional Formula

Alejandro Sanz-Paris, Diana Boj-Carceller, Beatriz Lardies-Sanchez, Leticia Perez-Fernandez and Alfonso J Cruz-Jentoft

**Table S1.** HDSF composition.

| **Approximate Analysis** | **Units** | **Per 100 mL** |
| --- | --- | --- |
| Energy | Kcal/kJ | 150/625 |
| Proteins | g | 7.5 |
| Calcium and sodium caseinate | % | 80 |
| Soy protein isolate | % | 20 |
| Carbohydrates | g | 12.75 |
| Fats | g | 7.5 |
| Saturated fatty acids | g | 0.53 |
| Monounsaturated fatty acids | g | 4.90 |
| Total dietary fibre | g | 1.50 |
| Prebiotics | g | 1.00 |
| Water | g | 76.96 |
| Taurine | mg | 15 |
| Carnitine | mg | 12 |
| Inositol | mg | 85 |
| Choline | mg | 60 |
| **Minerals** | | |
| Sodium | mg | 140 |
| Potassium | mg | 165 |
| Chloride | mg | 145 |
| Calcium | mg | 100 |
| Phosphorus | mg | 100 |
| Magnesium | mg | 31 |
| Iron | mg | 0.85 |
| Zinc | mg | 1.7 |
| Manganese | mg | 0.44 |
| Copper | mcg | 105 |
| Iodine | mcg | 15 |
| Selenium | mcg | 9.5 |
| Chromium | mcg | 9.0 |
| Molybdenum | mcg | 16 |
| **Vitamins** | | |
| Vitamin A | mcg RE | 144 |
| Vitamin D_3_ | mcg | 2.50 |
| Vitamin E | mg α TE | 3.02 |
| Vitamin K_1_ | mcg | 12 |
| Vitamin C | mg | 13 |
| Folic acid | mcg | 40 |
| Vitamin B_1_ | mg | 0.26 |
| Vitamin B_2_ | mg | 0.34 |

**Table S1.** *Cont.*

| **Approximate Analysis** | **Units** | **Per 100 mL** |
| --- | --- | --- |
| Vitamin B_6_ | mg | 0.39 |
| Vitamin B_12_ | mcg | 0.50 |
| Niacin | mg NE | 3.0 |
| Pantothenic acid | mg | 1.20 |
| Biotin | mcg | 7.2 |
